# The association between depression and sexual satisfaction among Malay elderly in Malaysia

**DOI:** 10.1016/j.heliyon.2019.e01940

**Published:** 2019-06-15

**Authors:** Hazwan Mat Din, Siti Aisyah Nor Akahbar, Rahimah Ibrahim

**Affiliations:** a Malaysian Research Institute on Ageing, Universiti Putra Malaysia, 43400, UPM, Serdang, Malaysia; b Faculty of Human Ecology, Universiti Putra Malaysia, 43400, UPM, Serdang, Malaysia

**Keywords:** Psychology

## Abstract

**Background:**

Malaysia is experiencing population ageing and expects to be an aged nation by 2030. Depression is one of the common disorder among elderly worldwide and the prevalence of depression in Malaysia is expected to increase as a result of population ageing. The association of depression and sexual satisfaction was unclear, particularly among the elderly. Therefore, this study aimed to examine the association between depression and sexual satisfaction among the Malay elderly population.

**Methods:**

One hundred and nine married community-dwelling elderly (Mean age = 63.23 years old) participated in this cross-sectional study. Hierarchical logistics regression was used to examine the association of depression on sexual satisfaction while controlling for potential confounders.

**Results:**

Prevalence of depression and sexual dissatisfaction were 26.6% and 20.2%, respectively. Depression was significantly associated with sexual satisfaction (P = 0.002; OR = 0.19, 95% CI = 0.06, 0.66). Depressed participants were 81% less likely to experience sexual satisfaction compared to those without depression.

**Limitations:**

Cross-sectional study design assessing the association between depression and sexual satisfaction.

**Conclusions:**

Findings from this study suggest that attention should be given to the prevention and treatment of depression among the elderly as a mechanism to improve sexual health.

## 1 Introduction

Depression is a common disorder that can affect overall health and quality of life of those suffering from it. It is expected to be the second leading cause of disability across the globe by 2020, particularly in women (Murray and Lopez, 1997). In Malaysia, it is reported that the prevalence of depression among elderly ranged from 6.3 to 18.0 % (Imran et al., 2009; Mohd Sidik et al., 2003; Mukhtar and P. S. Oei, 2011; Maniam et al., 2013). This prevalence is expected to increase as Malaysia is experiencing rapid population ageing and the country is expected to be an aged nation by 2030 (Samad and Mansor, 2017).

Depression influences an individual's emotion which then can result in many problems, mostly involving aspects of life including sexual functioning. Psychological and emotional health are important contributors to sexual functioning in adults (Offman and Matheson, 2005). Even though there is uncertainty of the association between depression and sexual satisfaction (Davison et al., 2009), depression is intimately correlated with sexual dissatisfaction (Kalmbach et al., 2012; Östman, 2008). Symptoms of depression such as fatigue and loss of pleasure could decrease sexual satisfaction (Suvak et al., 2012).

Studies among the elderly population shows mixed findings on the association between depression and sexual satisfaction. For example, a cross-sectional study among 169 heart failure elderly with a mean age of 64 years old showed present of depression contributed significantly toward sexual satisfaction when controlling for age, disease severity and use of antidepressant (Mosack et al., 2011). Similar findings were reported among prostate patients with mean age 67 years old (Garos et al., 2007). However, a recent study by (Scott et al., 2012) reported that depression was not significantly associated with sexual satisfaction among community-dwelling elderly. The study also reported depression decreased sexual satisfaction through communication as mediator.

In sum, a review of past study focusing on the association between depression and sexual satisfaction shows apparently conflating results, among elderly related population. Therefore, the impact of depression needs more clarification. This study aimed to study the association between depression and sexual satisfaction among married community-dwelling Malay elderly.

## 2 Methods

### 2.1 Study participants

Data of this cross sectional study were collected among married community-dwelling elderly in Kuala Lumpur, Malaysia. The study was conducted from December 2014 to February 2015 involving 12 community associations. The inclusion criteria of this study were i) elderly aged 60 years and above, ii) Malay and iii) able to understand and response to question asked orally. A face to face interview was conducted by trained enumerator. Ethical consideration was obtained from the Ethics Committee for Research Involving Human Subjects Universiti Putra Malaysia (JKEUPM). With a response rate of 90.3%, a total of 109 participants agreed to participate in this study.

### 2.2 Measures

#### 2.2.1 Sexual satisfaction

Participants were asked to response to an item; *Do you derive satisfaction from your physical contact?* The item was rated based on yes and no response. Physical contacts included sexual intercourse and other sexual contacts such as kissing, holding hands and touching.

#### 2.2.2 Depression

Malay version Geriatric depression scale (GDS) was used to measure depression. GDS was specifically developed for uses among elderly and was reported to have satisfactory construct validity and reliability (Teh and Hasanah, 2004). For this study, a cut off value of greater than 5 is indication of depression (Pocklington et al., 2015).

#### 2.2.3 Chronic medical conditions

Participants were asked to answer whether they have been told by doctor that they have any of this chronic medical condition; hypertension, diabetes, arthritis, heart disease and cataract? If participants answered having the conditions, they were asked whether they were treated by doctors for the conditions; only cases reported and treated by doctors were considered positive.

#### 2.2.4 Sociodemographic

Sociodemographic variables, including age category, gender, education level and smoking status were included in the analysis.

### 2.3 Statistical analysis

All analysis were conducted using IBM SPSS 22. Univariate analysis was done to assess distribution of study variables and to check for potential association between study variables and sexual satisfaction. In multivariate analysis, a 3-step hierarchical logistic regression was conducted to assess the impact of depression on sexual satisfaction after controlling for potential confounders. Depression was entered in the block 1 of the analysis, followed by addition of sociodemographic variables in the block 2. Chronic medical conditions were added in the third block (Block 3). To determine the goodness of fit of the model, Hosmer-Lemeshow test was used. All tests significance level was set at 0.05.

## 3 Results

The data consisted of 109 married community-dwelling Malay elderly, including 70 men and 39 women, with over 60% of the participants were age 60–64 years old. The mean age of the participants was 63.23 (Standard deviation = 4.21). Table 1 presents the univariate associations and distribution of study variables according to sexual satisfaction.

**Table 1:** Descriptive statistics and bivariate analysis between sexual satisfaction and study variable (n = 109).

Variable	Total	Sexual satisfaction				χ ^2^	*P*
		No	%	Yes	%		
Age category							
60–64	73	19	26.0	54	74.0	6.88^b^	0.017
65–69	31	6	19.4	25	80.6		
70+	5	4	80.0	1	20.0		
Gender							
Male	70	17	24.3	53	75.7	0.54^a^	0.463
Female	39	12	30.8	27	69.2		
Education							
Informal	6	3	50.0	3	50.0	4.28^b^	0.123
Primary	66	20	30.3	46	69.7		
Secondary above	37	6	16.2	31	83.8		
Smoking status							
Never smoked	55	16	29.1	39	70.9	0.33^a^	0.325
Stopped smoking	24	8	33.3	16	66.7		
Still smoking	30	5	16.7	25	88.3		
Hypertension							
No	31	9	29.0	22	71.0	0.13^a^	0.718
Yes	78	20	25.6	58	74.4		
Diabetes							
No	69	15	21.7	54	78.3	2.28^a^	0.131
Yes	40	14	35.0	26	65.0		
Arthritis							
No	76	14	18.4	62	81.6	8.61^a^	0.005
Yes	33	15	45.5	18	54.5		
Heart disease							
No	101	24	23.8	77	76.2	5.70^b^	0.030
Yes	8	5	62.5	3	37.5		
Cataract							
No	103	25	24.3	78	75.7	5.22^b^	0.042
Yes	6	4	66.7	2	33.3		
Depression							
No	87	16	18.4	71	81.6	14.90^a^	<0.001
Yes	22	13	59.1	9	40.9		

Abbreviation: % = Percentage; χ^2^ = Chi-square value.

a Chi-square test.

b Fisher's exact test.

The prevalence of having sexual satisfaction was 73.4% among study population. Prevalence sexual satisfaction were highest among 65–69 age group (80.6%), male (75.7%), having secondary and above education level (83.8%) and never smoked (70.9). Univariate analysis showed age, arthritis, heart disease, cataract and depression were significantly associated with sexual satisfaction.

### 3.1 Results of hierarchical logistic regression

Block 1 of the hierarchical logistic regression was highly significant, χ^2^ (1) = 13.48, *P* < 0.001), showed depression was significantly associated with sexual satisfaction without controlling for any potential confounders. Addition of sociodemographic variables in block 2 analysis showed the model was significant, χ^2^ (8) = 24.3, *P* = 0.002. Depression remained strongly significant after addition of sociodemographic variables as confounder. Addition of chronic medical conditions in block 3 was highly significant, χ^2^ (13) = 39.78, *P* < 0.001, as depression remained significant after controlling for all potential confounders in this study. Table 2 presents the results of hierarchical logistic regression. All blocks of hierarchical logistic regression showed model fit based in assessment of Hosmer-Lemeshow test (non-significant). As predicted, block 3 revealed depression was significantly associated with sexual satisfaction after controlling for age, gender, education, smoking status and chronic medical conditions. Thus, participants with depression were 81% less likely to experience sexual satisfaction from physical contacts compared to those without depression.

**Table 2:** Summary of hierarchical logistic regression.

Variable	Block 1			Block 2			Block 3		
	OR	95% CI		OR	95% CI		OR	95% CI	
		Lower	Upper		Lower	Upper		Lower	Upper
Depression	0.16^b^	0.06	0.43	0.16^b^	0.05	0.48	0.19^a^	0.06	0.66
Age category (Ref: 60–64)									
65–69				1.57	0.48	5.10	2.91	0.70	12.04
70+				0.13	0.01	1.56	0.11	0.01	1.86
Gender				1.21	0.25	5.89	1.04	0.17	6.43
Education (Ref: Informal)									
Primary				1.74	0.23	13.20	0.79	0.05	11.81
Secondary above				4.53	0.48	42.91	4.30	0.25	74.51
Smoking status (Ref: Never smoked)									
Stopped smoking				1.09	0.21	5.69	1.08	0.15	8.00
Still smoking				2.59	0.43	15.64	2.49	0.29	21.71
Hypertension							3.27	0.77	13.79
Diabetes							0.77	0.21	2.78
Arthritis							0.20^a^	0.06	0.66
Heart disease							0.11^a^	0.01	0.90
Cataract							0.53	0.04	6.72
χ^2^	13.48			24.30			39.78		
*df*	1			8			13		
Model significance	<0.001			0.002			<0.001		

Abbreviation: 95% CI = 95% Confidence interval; OR = Odds ratio.

a *P* ≤ 0.05.

b *P* < 0.001.

## 4 Discussion

The prevalence of community-dwelling elderly having sexual satisfaction in this population was 73.4%, as percentage was higher among men compared to women. In other words, this study revealed women experienced more sexual dissatisfaction compared to men. These findings are also reported by other study in the country (Sidi et al., 2007). For depression, overall prevalence of participants having depression in this study was 20.2%. World Health Organization reported prevalence of depression among elderly ranged from 10 to 20% (Srinivasa Murthy et al., 2001). These findings are also similar with study by Rashid and Tahir (2015), which reported 23.2% of married elderly experienced severe depression. However, some other studies reported higher prevalence population (Imran et al., 2009; Mohd Sidik et al., 2003; Mukhtar and P. S. Oei, 2011). Prevalence of depression in Malaysia varied due to uses of different instruments and cut off value to measure depression. Additionally, findings from this study showed among study participants who had depression, 59.1% of them were not satisfied with their sexual lives. The findings also showed greater number of depressed participants were experiencing sexual dissatisfaction.

Having depression is recognized as contributing to decreased libido among men and women of all ages (Perlman et al., 2007). In this study, the results of the 3-step hierarchical logistic regression revealed having depression were inversely associated with sexual satisfaction when sociodemographic variables and chronic medical conditions were controlled. This finding is consistent with other studies (Garos et al., 2007; Mosack et al., 2011; Nicolosi et al., 2004; Offman and Matheson, 2005; Steinke et al., 2008; Valadares et al., 2008). However, there are studies which reported that depression does not linked with sexual satisfaction (Scott et al., 2012; Shifren et al., 2008). For example, cross-sectional study conducted among married couples by Scott et al. (2012) showed that depression was not directly associated with sexual satisfaction for both gender. One may speculate that the inconsistent of findings from this study with other studies may be due to sociocultural differences of the study population.

Sexual satisfaction may also be explained by impact of depression through presence of health problems (Schulz et al., 2000). In women, a population-based survey found that depression increases the odds of sexual problems by more than 2 times when controlling for other confounders (Shifren et al., 2008). In men, depression has been found to be associated with sexual problems, which results in decreased sexual interest, arousal and sexual quality (Jagus and Benbow, 2002). Recent study in Malaysia has reported that elderly having sexual problems in form lack of interest, unpleasant sex and unable to orgasm during sex (Minhat et al., 2019a, Minhat et al., 2019b).

This study revealed arthritis and heart disease as significant confounders in the analysis. Similar finding also reported arthritis significantly associated with sexuality in elderly Minhat et al., 2019a, Minhat et al., 2019b). It is important to note that health-related issue including digestive disorder, respiratory problem and heart disease are associated with depression among elderly living in community (Mills, 2001). Both health problems and depression may negatively impact sexual functioning, especially sexual satisfaction.

The main limitation of the current study is its study design; cross-sectional design, which raises the issue for causal relationship between the variables studied. Secondly, the use a single item measure for sexual satisfaction. Due to lack of well-validated Malay version instrument to measure sexual satisfaction in local population, the single item measure was used. However, it noteworthy to mention that this item was derived based on the use of two item measures recommended by Laumann et al. (1994). The third limitation is the participants involved in this study were from Malay race, which is the majority race in Malaysia. So caution should be taken when generalizing this findings among Malaysian population. Fourth limitation is the current study does not explore the spouse's sexual function among the respondents. Local study among couples has reported there was a strong correlation between male and female sexual functioning (r = 0.42) (Yeoh et al., 2014). Further study in this area is highly recommended.

Despite the limitations of this study, the findings revealed depression is significantly associated with sexual satisfaction. Therefore, attention should be focused on depression prevention and treatment in later life.

## Declarations

### Author contribution statement

Mat Din Hazwan: Analyzed and interpreted the data; Contributed reagents, materials, analysis tools or data; Wrote the paper.

Nor Akahbar Siti Aisyah: Conceived and designed the experiments; Performed the experiments; Contributed reagents, materials, analysis tools or data.

Ibrahim Rahimah: Conceived and designed the experiments; Performed the experiments.

### Funding statement

This work was supported by Research University Grant Scheme, Universiti Putra Malaysia (Grant number: 04-04-11-1494RU).

### Competing interest statement

The authors declare no conflict of interest.

### Additional information

No additional information is available for this paper.
